# Somatic cancer mutations in the MLL1 histone methyltransferase modulate its enzymatic activity and dependence on the WDR5/RBBP5/ASH2L complex

**DOI:** 10.1002/1878-0261.12041

**Published:** 2017-03-10

**Authors:** Sara Weirich, Srikanth Kudithipudi, Albert Jeltsch

**Affiliations:** ^1^ Institute of Biochemistry Faculty of Chemistry University of Stuttgart Germany

**Keywords:** enzyme inhibition, enzyme regulation, histone methylation, MM‐102, protein lysine methyltransferase, somatic mutations

## Abstract

Somatic missense mutations in the mixed lineage leukemia 1 (MLL1) histone H3K4 methyltransferase are often observed in cancers. MLL1 forms a complex with WDR5, RBBP5, and ASH2L (WRA) which stimulates its activity. The MM‐102 compound prevents the interaction between MLL1 and WDR5 and functions as an MLL1 inhibitor. We have studied the effects of four cancer mutations in the catalytic SET domain of MLL1 on the enzymatic activity of MLL1 and MLL1–WRA complexes. In addition, we studied the interaction of the MLL1 mutants with the WRA proteins and inhibition of MLL1–WRA complexes by MM‐102. All four investigated mutations had strong effects on the activity of MLL1. R3903H was inactive and S3865F showed reduced activity both alone and in complex with WRA, but its activity was stimulated by the WRA complex. By contrast, R3864C and R3841W were both more active than wild‐type MLL1, but still less active than the wild‐type MLL1–WRA complex. Both mutants were not stimulated by complex formation with WRA, although no differences in the interaction with the complex proteins were observed. These results indicate that both mutants are in an active conformation even in the absence of the WRA complex and their normal control of activity by the WRA complex is altered. In agreement with this observation, the activity of R3864C and R3841W was not reduced by addition of the MM‐102 inhibitor. We show that different cancer mutations in MLL1 lead to a loss or increase in activity, illustrating the complex and tumor‐specific role of MLL1 in carcinogenesis. Our data exemplify that biochemical investigations of somatic tumor mutations are required to decipher their pathological role. Moreover, our data indicate that MM‐102 may not be used as an MLL1 inhibitor if the R3864C and R3841W mutations are present. More generally, the efficacy of any enzyme inhibitor must be experimentally confirmed for mutant enzymes before an application can be considered.

**Abbreviations**
CD: circular dichroism
MLL: mixed lineage leukemia
PKMT: protein lysine methyltransferase
PTM: posttranslational modification
WRA complex: WDR5, RBBP5, and ASH2L complex

## 1 Introduction

Histone posttranslational modifications such as methylation, phosphorylation, acetylation, and ubiquitination together with DNA methylation and noncoding RNAs establish the epigenetic code which regulates chromatin states (Bannister and Kouzarides, 2011; Bonasio *et al*., 2010; Jeltsch and Jurkowska, 2014; Margueron and Reinberg, 2010; Tan *et al*., 2011). Protein lysine methyltransferases (PKMTs) catalyze the methylation of lysine residues at the N‐terminal tails of histones (H3 and H4) and other proteins (Cheng *et al*., 2005; Clarke, 2013; Del Rizzo and Trievel, 2014; Dillon *et al*., 2005; Kudithipudi and Jeltsch, 2016; Zhang *et al*., 2015) and thereby play an important role in gene expression, cellular development and many diseases including cancer (Chi *et al*., 2010; Dawson and Kouzarides, 2012; Kudithipudi and Jeltsch, 2014). The Mixed lineage leukemia (MLL) PKMT family comprises MLL1‐4, SET1A, and SET1B, which are majorly involved in introducing H3K4 methylation in human cells and thereby play an important role in transcriptional regulation, particularly in early development and hematopoiesis (Krivtsov and Armstrong, 2007; Piunti and Shilatifard, 2016; Shilatifard, 2008; Volkel and Angrand, 2007; Zhang *et al*., 2013). The MLL paralogs vary in length and domain architecture and have nonredundant cellular functions. H3K4 can be mono‐, di‐, and trimethylated and H3K4me1 is located at active enhancers, whereas H3K4me3 is majorly present on active promotors. MLL1 is an intensively studied member of the MLL family and is essential for the control of developmentally regulated gene expression. Moreover, MLL1 misregulation is linked to acute lymphoid and myelogenous leukemia (Dou and Hess, 2008; Krivtsov and Armstrong, 2007; Muntean and Hess, 2012). MLL1 undergoes chromosomal translocation, where its N‐terminal part is fused to different partner proteins such as AF4 and AF9 generating oncoproteins, which further leads to deregulated expression of the HoxA9 and Meis1 genes.

MLL proteins contain a catalytically active SET [Su(var)3‐9, enhancer‐of‐zeste and trithorax] domain (Cheng *et al*., 2005; Dillon *et al*., 2005). In the majority of the SET domain PKMTs, such as Dim‐5, Set7/9, and Set8, the residues from the preSET, SET (including SET‐N, SET‐I, and SET‐C subdomains) and postSET regions form a catalytic channel that positions the substrate lysine side chain in an appropriate chemical environment for methyl transfer. However, MLL1 has a distinct SET domain conformation, in which the SET‐I region orients differently than in Dim‐5 resulting in an open structure, which cannot facilitate the proper alignment of target lysine and cofactor (Southall *et al*., 2009). Because of this, the isolated MLL1 protein exhibits only weak H3K4 methylation activity (Dou *et al*., 2006; Patel *et al*., 2009). MLL proteins form large complexes in the cell, together with the tryptophan‐aspartate repeat protein‐5 (WDR5), retinoblastoma‐binding protein‐5 (RBBP5), and absent small homeotic‐2‐like (ASH2L) proteins (WRA) (Dou *et al*., 2006; van Nuland *et al*., 2013; Patel *et al*., 2009; Steward *et al*., 2006). Interaction of the MLL1 SET domain with the WRA proteins reorients its SET‐I region, leading to a closed conformation which is active. While this effect is mainly due to the interaction of the RA heterodimer with MLL1, it has been found that MLL1 requires all the three complex partners (WDR5, ASH2L, RBBP5) to exhibit the maximal methyl transferase activity (Cao *et al*., 2010; Li *et al*., 2016; Southall *et al*., 2009). This is in contrast to the MLL1 homologs MLL2, MLL3, and MLL4 which do not require WDR5 for the optimal activity (Li *et al*., 2016). The reason for this difference is that the MLL1‐RA interaction is weaker than the interaction of other MLL family members with RA and MLL1‐RA complex formation depends on the presence of WDR5 as bridging partner. WDR5 interacts with the WDR5 interaction motif (WIN) in MLL1 (Patel *et al*., 2008) and this additional interaction is important for the stabilization of the MLL1–WRA complex and for the maximal methyl transferase activity (Avdic *et al*., 2011; Li *et al*., 2016). Depletion of WDR5 reduces the H3K4 methylation in cells and also decreases the expression of Hox genes (Wysocka *et al*., 2005). In addition, increased expression of MLL1 and WDR5 is observed in ALL suggesting that WDR5 exhibits its oncogenic effect through MLL1 by increasing H3K4 methylation (Ge *et al*., 2016). Several small molecule inhibitors were designed to disrupt the MLL1‐WDR5 interaction as a novel therapeutic strategy to treat leukemia caused by MLL1 hyperactivity (Cao *et al*., 2014; Karatas *et al*., 2013; Li *et al*., 2016).

Apart from chromosomal translocations, several cancers contain somatic mutations in MLL1, which include nonsense, missense, and frameshift mutations (Kudithipudi and Jeltsch, 2014). Interestingly, ignoring silent mutations, the mutational spectrum of MLL1 retrieved from COSMIC in Jan. 2017 shows 78% missense mutations and only 22% nonsense mutations and frameshifts, suggesting that the missense mutations may cause gain‐of‐function phenotypes. Twenty‐three residues with missense mutations are located in the SET domain of the enzyme, where they could directly affect its methyltransferase activity or substrate specificity. Somatic cancer mutations in MLL1 have not yet been studied, but recently germline mutations in MLL2 that were observed in Kabuki syndrome were investigated in the context of MLL1 (Shinsky *et al*., 2014). It was the aim of our work to investigate if selected missense mutations in the SET domain of MLL1 change its enzymatic properties.

**Figure 1:**
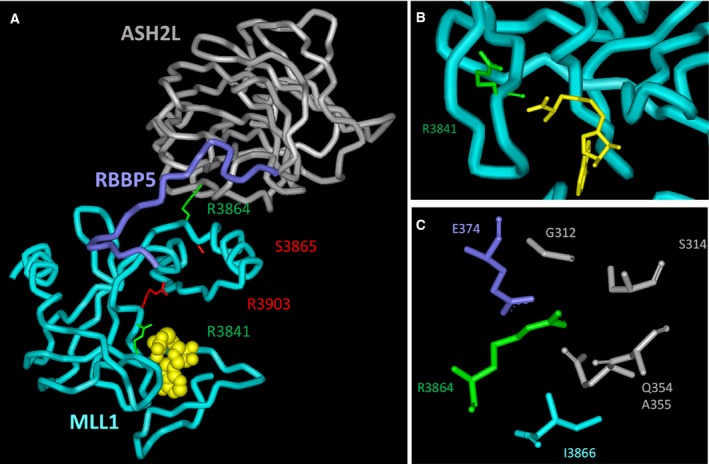
MLL1 mutations investigated in this study. (A) Crystal structure of the MLL1‐SET domain N3861I/Q3867L variant in complex with RBBP5 (residue 330–360) and ASH2L (residue 286–505) bound to cofactor product *S*‐adenosyl‐l‐homocysteine (AdoHcy) (pdb code: 5F6L) (Li *et al*., 2016). The MLL1 protein is shown in cyan, RBBP5 in blue and ASH2L in gray. The cofactor product AdoHcy is visualized in yellow. The S3865 and R3903 exchange of which we show here to cause loss of activity are displayed in red. The R3864 and R3841 residues exchange of which we show here to stimulate the methyltransferase activity are displayed in green. (B) Detailed view of R3841 (green), which forms a main‐chain NH contact to the carboxylic acid moiety of the cofactor product AdoHcy (yellow). (C) R3864 (green) forms an interface to ASH2L (residues G312, S314, Q354, A355 in gray) and RBBP5 (residue E374 in blue).

At the time of the design of this study in 2013, four mutations in the SET domain of MLL1 were selected (R3841W, R3864C, S3865F and R3903H) for experimental investigation, because they are located next to functional regions of MLL1 like the peptide or AdoMet binding sites or putative complex partner interaction sites (Fig. 1A–C). R3841 is located in the SET‐N part of the MLL1 SET domain next to the active center and it is engaged in a main‐chain H‐bond to the carboxylate moiety of AdoMet, while its side chain points toward the SET‐I domain and RBBP5. An Arg is conserved at this position in the MLL1/2/TRX subfamily of MLL enzymes, while MLL3/4 and SET1A/B contain Leu and Trp, respectively. R3864 and S3865 are located in the SET‐I domain in the loop contacting ASH2L. R3864 participates in the interface with RBBP5 and ASH2L and Arg is conserved at this position in all MLL proteins. S3865 is not directly involved in the ASH2L and RBBP5 interface and it is only conserved in the MLL1/2/TRX subfamily of MLL enzymes, other subfamilies contain Thr or Gln at this position. R3903 is located in the SET‐C part of the MLL1 SET domain connecting the SET‐C and SET‐I subdomains. It is fully conserved among all MLL enzymes and could also be involved in contacting RBBP5. The selected mutations were found in different cancers, *viz*. R3841W in prostate cancer (Barbieri *et al*., 2012), R3864C in lung cancer (Network, 2012), S3865F in skin cancer (Durinck *et al*., 2011), and R3903H in large intestine cancer (Network, 2012).

We observed that two of the four selected somatic cancer mutants, R3864C and R3841W, increased the catalytic activity compared to wild‐type MLL1, whereas two other mutations, S3865F and R3903H, caused a reduction or loss of activity. Strikingly, our data demonstrate that the R3841W and R3864C mutants behave differently with respect to complex partner requirement than wild‐type MLL1, because they exhibit their maximal methyltransferase activity without the complex partners and were not stimulated further by the complex formation. This indicates that these somatic cancer mutations in MLL1 induce local conformational changes in the SET domain, which increase the methyltransferase activity and abrogate complex partner dependency. This presumably leads to changes in the cellular MLL1 activity, because the mutant enzymes have lost activity control by the WRA complex. From a therapeutic point of view, we show that MLL1‐WDR5 interaction inhibitors are likely less useful for cancers containing these MLL1 mutations.

## 2 Materials and methods

### 2.1 Cloning, expression, and purification of proteins and protein variants

The DNA encoding the SET domain of MLL1 (also called KMT2A) (amino acids 3745–3969 of Q03164) was amplified from cDNA isolated from HEK293 cells and cloned into pGEX‐6p2 as GST‐fusion protein. MLL1 somatic cancer mutations located in the SET domain of MLL1 were cloned using a megaprimer PCR mutagenesis protocol. For protein expression, *Escherichia coli* BL21‐DE3 codon plus cells were transformed with the corresponding plasmid and grown in Luria–Bertani media at 37 °C until they reached 0.6 to 0.8 OD_600_. Afterward, the cells were shifted to 20 °C for 10 min and then induced overnight with 1 mm isopropyl‐beta‐d‐thiogalactopyranoside. The next day, cells were harvested by centrifugation (5000 ***g***). Protein purification of the GST‐fusion protein was conducted as described before (Dhayalan *et al*., 2011). The complex proteins WDR5, RBBP5, and ASH2L were expressed and purified as described (Avdic *et al*., 2011).

### 2.2 Circular dichroism analyses of the purified MLL1 SET domain proteins

Circular dichroism (CD) measurements were performed at 22 °C as described using a J‐815 circular dichroism spectrophotometer (JASCO Corporation, Tokyo, Japan) (Weirich *et al*., 2015). For CD melting temperature determination, the MLL1 SET domains were diluted in 200 mm KCl to a final concentration of 20 μm. The CD signal was measured at a wavelength of 210 nm in a 0.1‐mm cuvette in the temperature range from 20 °C to 80 °C applying a temperature increase of 1 °C·min^−1^. The melting temperature was determined using the instrument software.

### 2.3 *In vitro* peptide methylation by plate assay

For peptide methylation, a microplate assay was used basically as described (Gowher *et al*., 2005). MLL1‐SET (0.8 μm) was incubated in the absence or presence of equimolar amounts of complex proteins with 0.625 μm biotinylated H3 (1–19) peptide (Intavis) in methylation buffer (50 mm Tris/HCl pH 8, 200 mm NaCl, 5 mm MgCl_2_, and 3 mm DTT) containing 0.76 μm radioactively labeled [methyl‐^3^H]‐AdoMet (PerkinElmer Life Sciences, Boston, MA, USA) for 3 h at 22 °C in an Eppendorf tube. Afterward, the samples were transferred to an avidin‐coated microplate (Greiner, Bio‐One, Frickenhausen, Germany) and shaken for 30 min. To remove unbound peptide, the microplate was washed with 1× PBST and 500 mm NaCl. For elution of the bound peptide, 50 mm HCl was added and incubated for 1 h. The released radioactivity was analyzed by liquid scintillation counting in a Hidex 300SL (HIDEX, Mainz, Germany).

### 2.4 Histone protein methylation assay

Protein methylation was performed by incubating 1.6 μm recombinant H3.1 (New England Biolabs, Frankfurt, Germany) with 0.56 μm MLL1‐SET in the presence or absence of equimolar amounts of complex partners WDR5, RBBP5, and ASH2L in methylation buffer containing 50 mm Tris/HCl pH 8, 200 mm NaCl, 5 mm MgCl_2_, 3 mm DTT, and 0.76 μm radioactively labeled [methyl‐^3^H]‐AdoMet (PerkinElmer Life Sciences) for 2 h at 22 °C. For inhibitor studies, 0.4 μm MM‐102 (EMD Millipore compound 5.00649.0001, Merck, Chemicals Gmbh, Darmstadt, Germany) was included. Methylation reactions were stopped by adding SDS loading buffer and heating of the samples to 95 °C for 5 min. Then, the samples were separated on a 16% SDS/PAGE gel and the methylation signal was detected by autoradiography after soaking the gel with Amplify solution (GE Healthcare, Buckinghamshire, UK).

### 2.5 WDR5–MLL1 interaction assay

To study the WDR5–MLL1 interaction by GST pull‐down, 0.56 μm of GST‐tagged MLL1‐SET and equimolar amounts of His‐tagged WDR5 were incubated with or without 0.1 mm inhibitor MM‐102 in incubation buffer (25 mm Tris/HCl pH 8, 5 mm MgCl_2_, 100 mm KCl, 10% glycerol, 0.1% NP‐40, and 200 μm PMSF) for 30 min at 4 °C. As negative control, a reaction with same amounts of GST was conducted. Afterward, samples were bound to glutathione–Sepharose™ 4B beads (GE Healthcare) and incubated for 30 min. In the next step, the beads were washed two times with washing buffer 1 (25 mm Tris/HCl pH 8, 5 mm MgCl_2_, 300 mm KCl, 10% glycerol, 0.1% NP‐40, and 200 μm PMSF), two times with washing buffer 2 (25 mm Tris/HCl pH 8, 5 mm MgCl_2_, 500 mm KCl, 10% glycerol, 0.1% NP‐40, and 200 μm PMSF) and once with incubation buffer. Finally, the supernatant was incubated in SDS loading buffer for 5 min at 95 °C and the samples analyzed on 16% SDS/PAGE gel.

### 2.6 MLL1‐RBBP5/ASH2L and MLL1‐WDR5/RBBP5/ASH2L interaction assays

To study the interaction of MLL1 with the RBBP5/ASH2L or WDR5/RBBP5/ASH2L complexes with AlphaScreen assays, 0.5 μm His‐tagged RBBP5 protein, 0.5 μm His‐tagged ASH2L protein and (if needed) 0.5 μm His‐tagged WDR5 were pre‐incubated for 30 min at 4 °C to form the corresponding complexes. A 10 μL aliquot of the complexes was loaded in each well of a microplate (1/2 Area plate™‐96; PerkinElmer). Then, 10 μL of 0.5 μm GST‐tagged MLL1‐SET was added and incubated for 1 h at 22 °C. Afterward, 0.8 μg nickel‐chelate acceptor beads (PerkinElmer) and 0.8 μg glutathione donor beads (PerkinElmer) were added and incubated for another 1 h in the dark at 22 °C. As negative controls, empty beads or beads incubated with 0.5 μm GST protein were included. The AlphaScreen light signal was measured with an EnSpire™ 2300 Multimode reader (PerkinElmer). The experiments were conducted in AlphaLISA Universal Buffer (PerkinElmer AL001C) containing PBS (10 mm phosphate, 137 mm NaCl, 2.7 mm KCl) pH 7.2, 0.1% BSA, and 0.01% Proclin‐300.

### 2.7 Quantitative analysis and statistics

Methylation signals were quantified by densitometry from autography films. For this, films with different exposure times were prepared to ensure that no signal saturation occurred. All experiments were conducted in biological replicates as indicated. Data are reported as averages and standard deviations of the mean (SEM). *P*‐values for all experiments are listed in Table S1.

## 3 Results

### 3.1 Cloning and purification of MLL1 mutants

**Figure 2:**
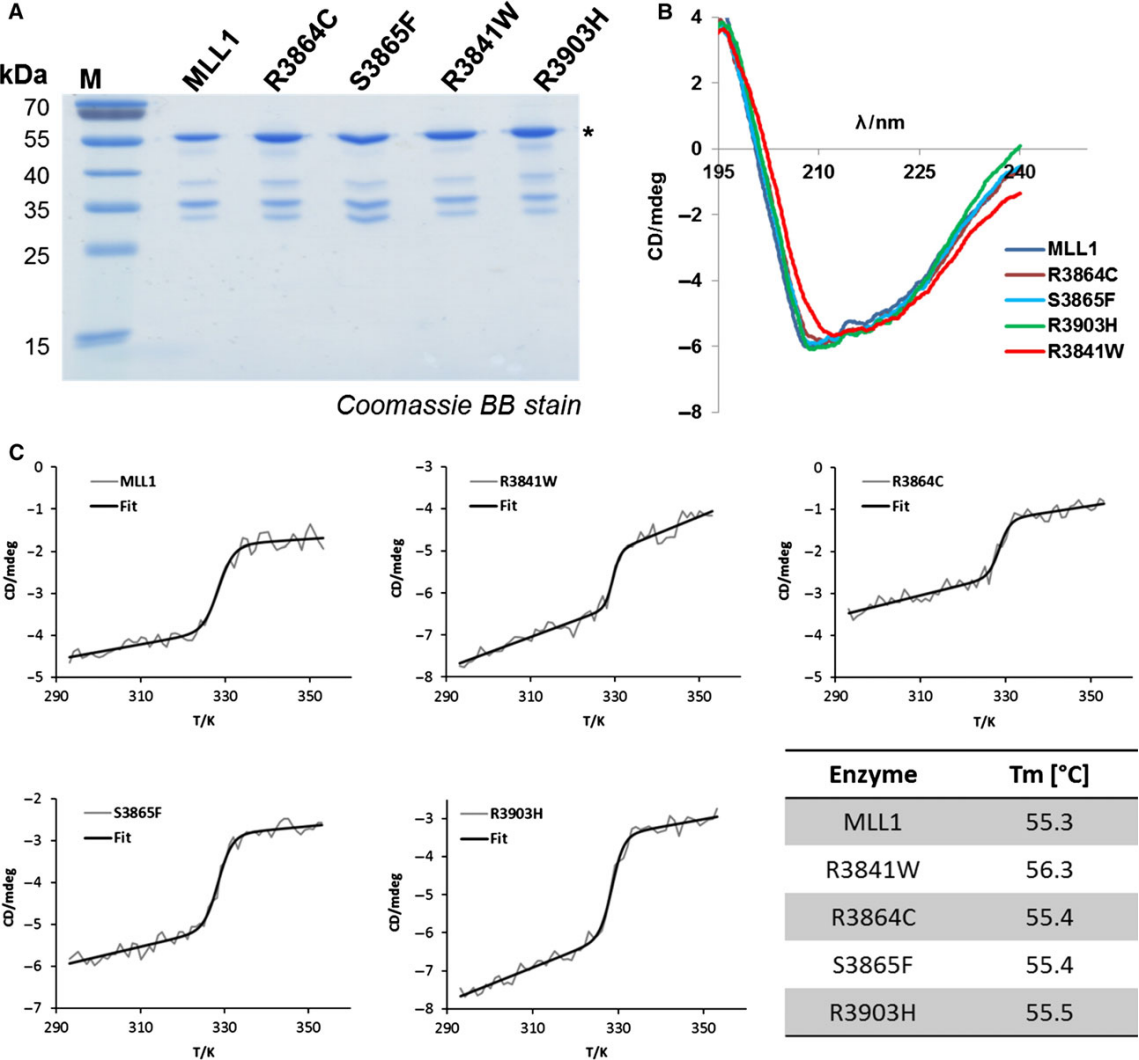
Protein purification and CD analyses of MLL1 mutants. (A) Coomassie BB‐stained SDS/polyacrylamide gel of the purified GST‐MLL1 cancer variants. All MLL1 mutant proteins were purified in comparable quality. (B) Circular dichroism spectra of purified MLL1 wild‐type and cancer variants R3864C, S3865F, R3841W, and R3903H. The figure shows average data of three independent measurements of independent protein preparations. MLL1 wild‐type and all cancer mutant proteins showed similar CD spectra, except R3841W which could be due to changes in conformation, folding or aggregation. (C) CD melting analysis of purified MLL1 wild‐type and cancer variants R3864C, S3865F, R3841W, and R3903H. Corresponding melting temperatures are listed in the table.

Recent exomic and genomic sequencing of cancer cells identified several somatic mutations in various histone PKMTs including MLL1 (Kudithipudi and Jeltsch, 2014). It was the aim of the current study to investigate the effects of somatic mutations in the SET domain of MLL1 (KMT2A) on its enzymatic properties. From the COSMIC database, four mutations in the SET domain of MLL1 were selected that are located next to functional regions including the peptide, AdoMet or complex partner interaction sites (Fig. 1A–C). The GST‐tagged SET domain of human MLL1 wild‐type and mutants were cloned, overexpressed in *E. coli* and purified by affinity chromatography in comparable quality (Fig. 2A). The secondary structure composition of the purified MLL1 mutant proteins was analyzed by circular dichroism spectroscopy (CD) (Fig. 2B). The R3864C, S3865F, and R3903H variants showed similar CD spectra as MLL1 wild‐type, which indicates that the wild‐type and mutant proteins are similarly folded. R3841W displayed a slight difference in the CD spectra, which indicates some changes in conformation, folding or aggregation state. To investigate the effect of the mutations on protein stability, CD melting experiments were conducted (Fig. 2C). MLL1 wild‐type and the three cancer mutants R3864C, S3865F, and R3903H revealed an identical melting temperature Tm = 55.4 (± 0.1) °C, while R3841W showed an increased melting temperature of 56.3 °C.

### 3.2 Catalytic activity of MLL1 mutants

**Figure 3:**
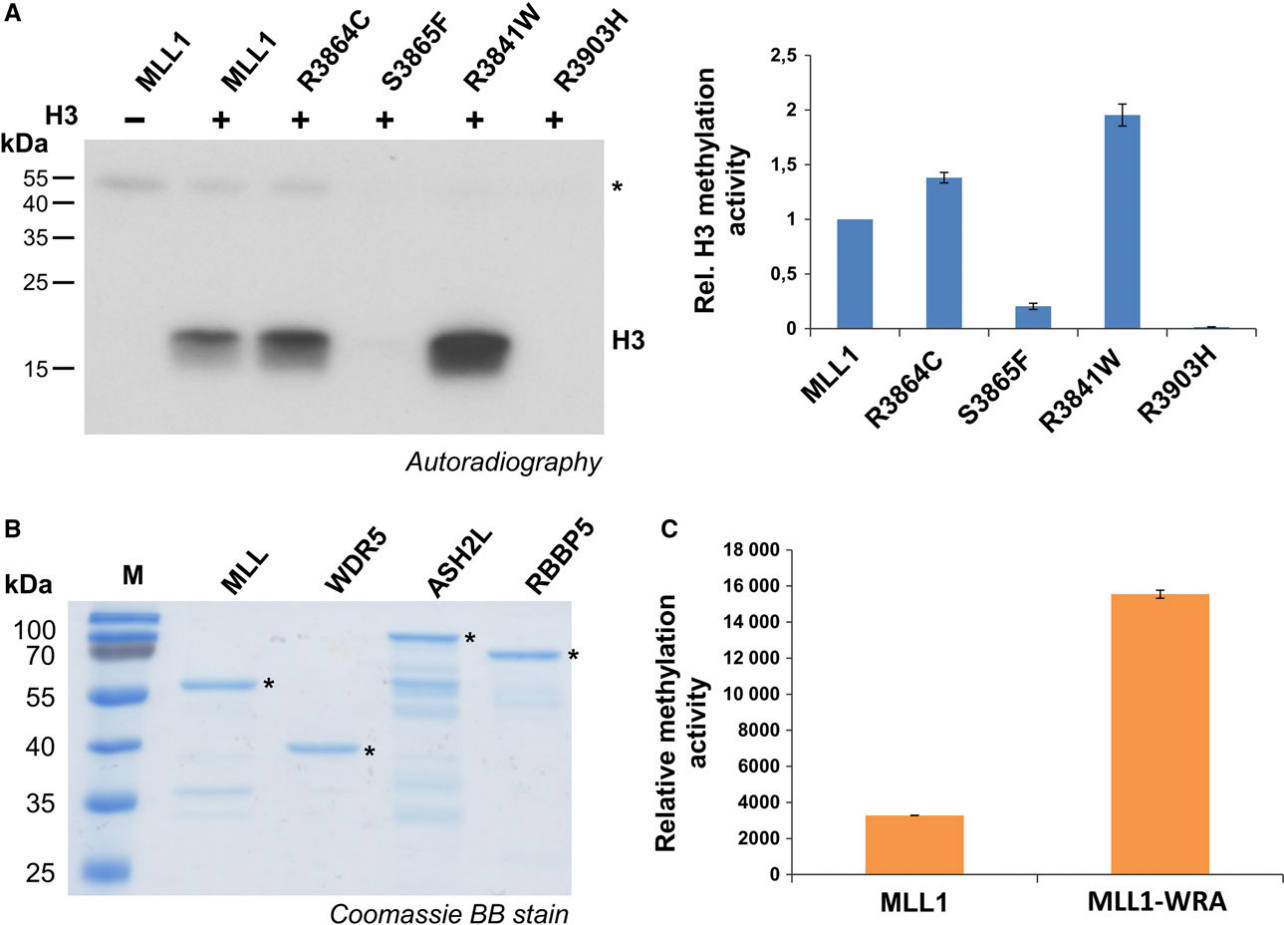
Catalytic activity of MLL1 mutants (A) Methylation of recombinant histone H3 by MLL1 wild‐type and cancer variants using radioactively labeled AdoMet. The left panel represents an autoradiographic image of an SDS/polyacrylamide gel showing H3 methylation signals obtained with MLL1, R3864C, and R3841W, whereas no methylation signal was detected for S3865F an R3903H. As negative control, MLL1 wild‐type without H3 substrate was used. The methylation signal of H3 is indicated. * indicates automethylation of MLL1. The right panel shows a quantitative analysis of the average of the H3 methylation observed in two experiments. Error bars indicate the standard error of the mean. (B) Coomassie BB‐stained SDS/polyacrylamide gel of the purified GST‐MLL1 together with equimolar amounts of His‐WDR5, GST‐ASH2L, and His‐RBBP5. (C) Methylation of histone H3 1–19 peptide by MLL1 or MLL1 in complex with the WRA proteins. Biotinylated H3 peptide was incubated with either isolated MLL1 wild‐type protein or together with equimolar amounts of the WRA complex in the presence of radioactively labeled AdoMet. The transfer of radioactively labeled methyl groups to the peptides was detected by liquid scintillation counting, and data were averaged from two independent experiments. The error bars indicate the standard error of the mean.

The activity of the isolated MLL1 variants was assessed by a radiometric histone H3 methylation assay. MLL1 wild‐type and the somatic variants were incubated with equal amounts of recombinant H3 in the presence of radioactively labeled AdoMet as cofactor. Comparable amounts of the MLL1 protein variants were used in the assay as illustrated in Fig. 2A. The methylated samples were separated using SDS/PAGE and the transfer of radioactively labeled methyl groups to histone H3 was detected by autoradiography (Fig. 3A). The results revealed that two of the mutants (R3864C, R3841W) were more active than the wild‐type protein (R3841W approximately twofold, R3864C approximately 1.5‐fold). In contrast, the other two mutants showed a strongly reduced activity (S3865F, approximately fivefold reduction) or were inactive within the detection range of the methylation assay (R3903H). Our data are in principal agreement with a previous study reporting that R3864Q is catalytically active and R3903T is inactive (Shinsky *et al*., 2014).

### 3.3 Catalytic activity of MLL1 mutants in complex with the WRA proteins

As MLL1 exhibits full methyltransferase activity only in the presence of the WRA complex, we purified the complex partner proteins (Fig. 3B) to investigate their stimulatory effect on MLL1 methyltransferase activity *in vitro*. MLL1 wild‐type was incubated with biotinylated H3 (1–19) peptide in the absence or presence of equimolar amounts of WRA complex using radioactively labeled AdoMet as cofactor. After purification of the peptides on avidin plates, the transfer of radioactively labeled methyl groups to the peptides was detected by scintillation counting. As expected a strong (about fivefold) stimulatory effect was detected after the addition of complex partners (Fig. 3C). Using the same expression constructs and peptide substrates, Avdic *et al*. (2011) observed a 15‐fold stimulation (Avdic *et al*., 2011). However, Avdic *et al*. used 5 μm MLL1 and WRA complex members, while we used only 0.8 μm. Therefore, MLL1–WRA complex formation was less complete in our experiment which can explain the threefold discrepancy between the levels of stimulation.

**Figure 4:**
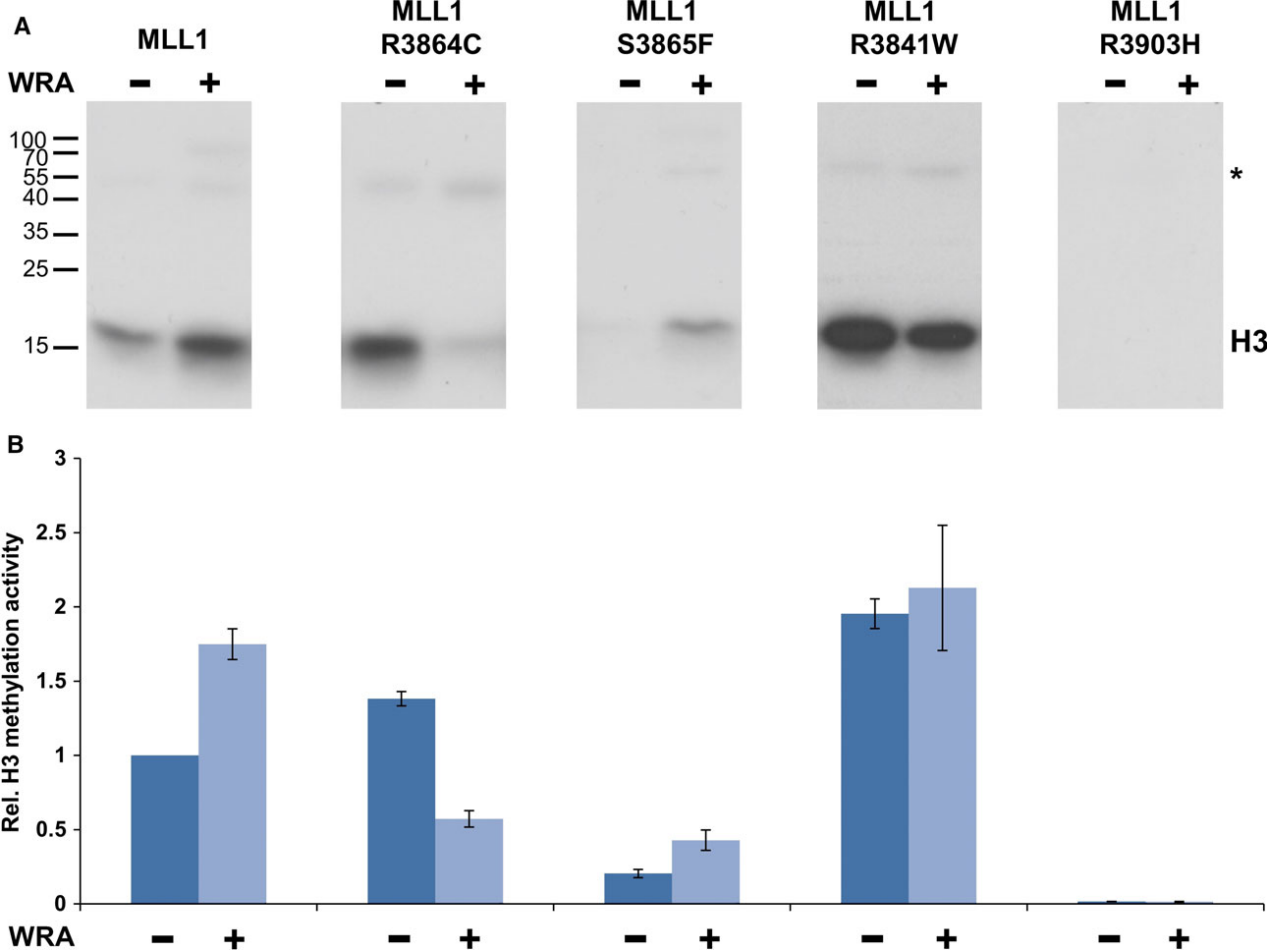
Methylation activity of MLL1 cancer variants in complex with WRA proteins. Recombinant H3 was methylated by MLL1 and mutant proteins in the absence or presence of the WRA complex. (A) Examples of autoradiographic image of an SDS/polyacrylamide gels. Samples with (+) or without (−) complex partners were loaded next to each other. The methylation signal of H3 is indicated. * represents automethylation of MLL1. (B) Quantitative analysis of the H3 methylation signal using duplicate experiments. The activity of isolated MLL1 was set to 1 and the other signals were normalized accordingly. Error bars indicate the standard error of the mean.

We next tested the activity of MLL1 wild‐type and cancer mutants in the presence of the complex members using H3 protein as substrate. MLL1 wild‐type and mutant proteins were incubated with equimolar amounts of complex partners in the presence of recombinant histone H3 and radioactively labeled AdoMet. Simultaneously, methylation assays were performed with the isolated MLL1 proteins for comparison. Samples with and without complex partners were loaded next to each other on SDS/PAGE gels and the transfer of radioactively labeled methyl groups was detected as described above (Fig. 4). In agreement with published data (Southall *et al*., 2009) and the peptide methylation experiments described above, MLL1 exhibited higher methyltransferase activity in the presence of the WRA complex members. S3865F was stimulated by the WRA complex to a similar degree as wild‐type MLL1, albeit at a lower overall activity level. R3903H remained inactive even in the presence of the WRA complex. Interestingly, R3864C and R3841W, which were more active than wild‐type MLL1 as isolated proteins, were not stimulated in the WRA complex or even showed a reduced activity in the complex (R3864C). This result is in agreement with a report showing that the R3864Q mutant displays a reduced activity in the presence of complex partners (Shinsky *et al*., 2014).

### 3.4 WDR5 binding to MLL1 proteins

As described above, the R3864C and R3841W mutants responded to the addition of the WRA complex members differently than the wild‐type. As the R3864C mutation is close to the WDR5 interface of MLL1, we investigated the interaction of the MLL1 mutants with WDR5 by GST pull‐down assays. For these experiments we also employed the MM‐102 compound, which mimics the GSARAE residues of the Win motif in MLL1 and competes for binding to WDR5. Thereby, it disrupts the interaction between WDR5 and MLL1 and inhibits the MLL1 methyltransferase activity (Karatas *et al*., 2013). MM‐102 has been described as an efficient drug that selectively inhibits the cell growth and initiates apoptosis in the cells harboring MLL1 fusion proteins. In addition, it was also shown to reduce the expression of MLL1 target genes such as HoxA9 and Meis1 in leukemia cell lines.

**Figure 5:**
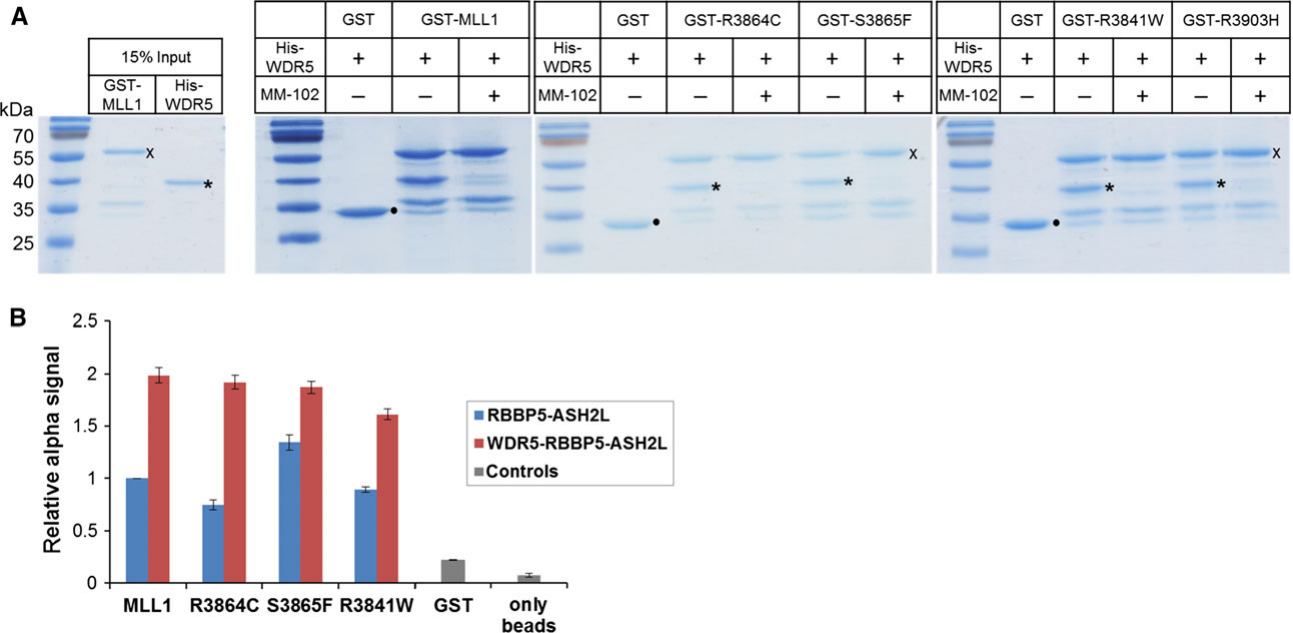
WDR5 and RBBP5/ASH2L binding by MLL1 proteins. (A) To investigate the interaction between MLL1 proteins and WDR5, GST pull‐down assays were performed. GST‐fused MLL1 proteins were incubated with His‐tagged WDR5 in the presence or absence of the MM‐102 inhibitor, which disrupts the interaction between WDR5 and MLL1. As control, 15% of the input was loaded on a separate SDS/polyacrylamide gel. ● indicates the bands corresponding to GST control; * indicates WDR5 bands; × indicates MLL1 bands (additional bands are degradation products of MLL1). (B) Interaction between the RBBP5/ASH2L or WDR5/RBBP5/ASH2L complexes and MLL1 wild‐type and variants analyzed using AlphaScreen assay. His‐tagged RBBP5/ASH2L or WDR5/RBBP5/ASH2L complexes were bound to nickel‐chelate acceptor beads and GST‐fused MLL1 mutant proteins were bound to glutathione donor beads. Beads with GST protein and empty beads were included as negative controls. The production of a light signal indicates the complex formation between RBBP5/ASH2L or WDR5/RBBP5/ASH2L and MLL1. The error bars indicate the standard error of the mean of four measurements.

GST‐fused MLL1 proteins were incubated with His‐tagged WDR5 in the presence or absence of MM‐102 and bound to GST beads. After several washing steps, the GST beads were boiled in SDS loading buffer and the samples were separated on SDS/PAGE. As shown in Fig. 5A, WDR5 interacted with all MLL somatic variants in the absence of MM‐102. However, in the presence of inhibitor, the protein band corresponding to WDR5 disappeared. This result indicates that none of the investigated mutations in MLL1 disrupts the WDR5 interaction and the MLL1‐WDR5 interaction remained responsive to inhibition by MM‐102 in all cases.

### 3.5 RBBP5/ASH2L or WDR5/RBBP5/ASH2L binding to MLL1 proteins

Our data showed that the MLL1 mutants responded differently to the addition of the WRA complex partners, but all the MLL1 variants interact with WDR5. Recently, Li *et al*. (2016) showed that MLL proteins primarily interact with the RBBP5/ASH2L heterodimer and WDR5 only serves to enhance this binding (Li *et al*., 2016). Therefore, we next analyzed the interaction between the RBBP5/ASH2L heterodimer or WDR5/RBBP5/ASH2L complex and MLL1 variants using AlphaScreen assays. His‐tagged RBBP5/ASH2L heterodimer or WDR5/RBBP5/ASH2L complex was bound to nickel‐chelate acceptor beads and GST‐fused MLL1 mutant proteins were bound to glutathione donor beads. Beads with GST protein and empty beads were included as negative controls. By complex formation between RBBP5/ASH2L or WDR5/RBBP5/ASH2L complex and MLL1, the acceptor beads are brought into proximity to the donor beads, which results in the production of a light signal. As shown in Fig. 5B, a comparable AlphaScreen signal was observed for MLL1 wild‐type and all cancer variants, which is indicative of a similar interaction of all MLL1 proteins with the RBBP5/ASH2L heterodimer. The AlphaScreen signal was increased for all MLL1 proteins when WDR5/RBBP5/ASH2L was used. This indicates that in each case the WRA interaction was stronger than the RA interaction, in agreement with the expectation that WDR5 further stabilizes the interaction between MLL1 protein variants and RBBP5/ASH2L heterodimer. In summary, the interaction between MLL1 and WDR5, MLL1 and RBBP5/ASH2L and also MLL1 and WDR5/RBBP5/ASH2L could be detected, which means that differences in the effects of WRA complex formation on the catalytic activity of the MLL1 mutants were not due to a loss of the interaction with the complex partners.

### 3.6 Effects of individual complex members on MLL1 activity

To further dissect the consequences of the MLL1 mutations, we determined the effects of all individual binary and tertiary interactions between MLL1 and its interaction partners on MLL1's catalytic activity. With wild‐type MLL1, no big changes in methylation signals were observed upon addition of any of the single protein partners or heterodimers (apart from a mild inhibition by RBBP5 alone). However, the addition of all three complex partners (WDR5, RBBP5, and ASH2L) caused a strong stimulation. The S3865F mutant showed a very similar profile at an overall roughly fivefold reduced activity level.

**Figure 6:**
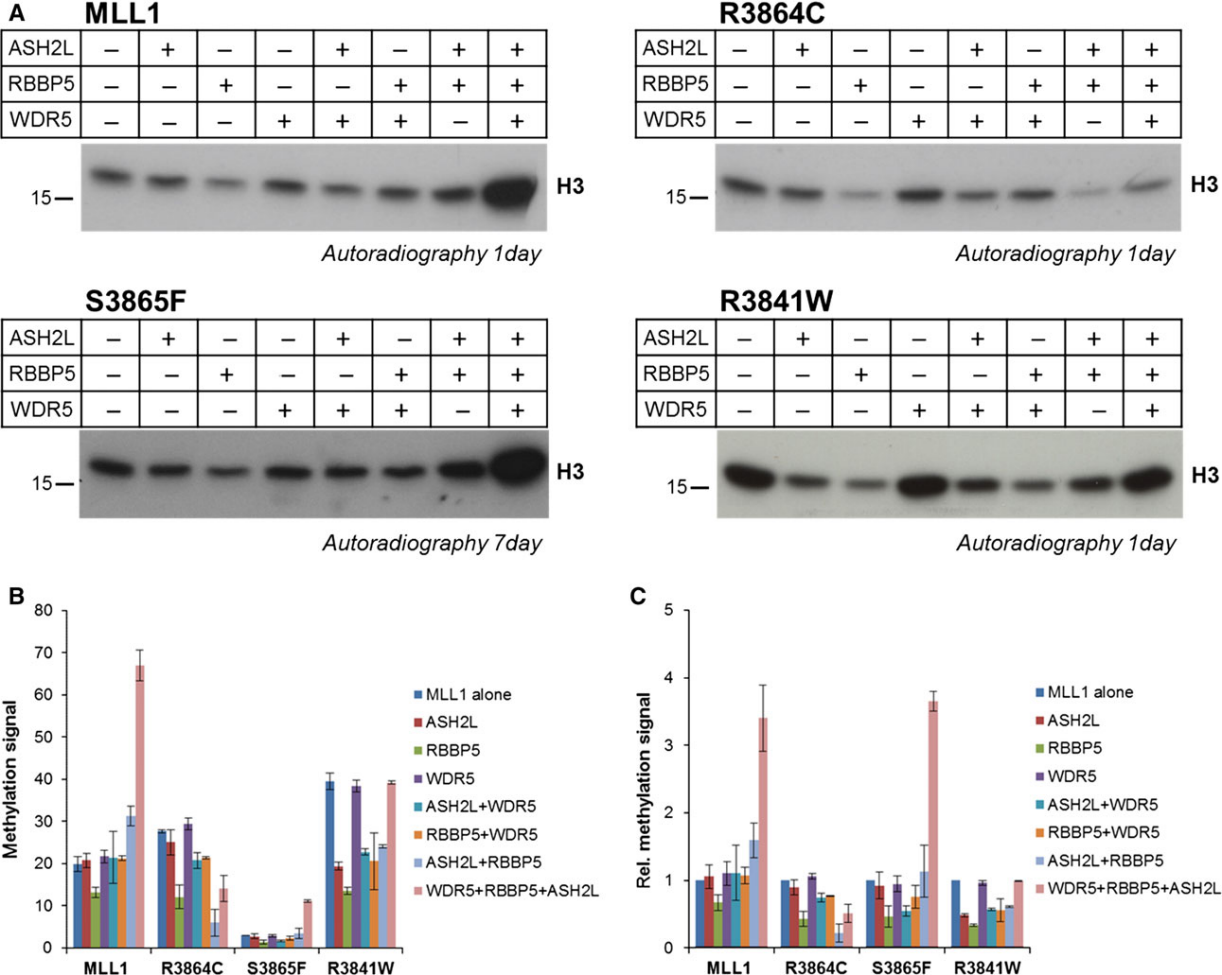
Effects of individual complex members on MLL1 activity. (A) Examples of H3 methylation assays to analyze the effects of all individual binary and tertiary interactions between MLL1 and its interaction partners on catalytic activity. The figure shows autoradiographic images of SDS/polyacrylamide gels. The methylation signal of H3 is indicated. (B) Quantitative analysis of absolute signal intensities of duplicate experiments. (C) The signals obtained from MLL1 mutant proteins in the absence of complex proteins was set to 1, and the other signals were normalized accordingly. The error bars in B and C indicate the standard error of the mean of two independent experiments.

The effects of most binary and tertiary complex partner interactions of the R3864C mutant resemble that of wild‐type MLL1. However, with this mutant a strong inhibition was observed after adding the RBBP5/ASH2L heterodimer. The addition of WDR5 to the R3864C‐RA complex caused an increase in activity that was slightly less pronounced than with wild‐type MLL1. Still, the final activity of the R3864C‐WRA complex was much lower than that of wild‐type MLL1–WRA, because of the lower activity level of R3864C‐RA. Hence, the reduced activity of R3864C‐WRA is mainly caused by the strong reduction in activity after the addition of RA. The profile of the R3841W mutant was considerably different. This mutant was highly active without any of the interaction partners, and the addition of RBBP5, ASH2L, or RA complex reduced the activity. Only WDR5 had no inhibitory effect. The addition of WDR5 to the R3841W‐RA complex brought activity back to the level of the free R3841W protein (Fig. 6).

### 3.7 Inhibition of the MLL1 proteins by MM‐102

**Figure 7:**
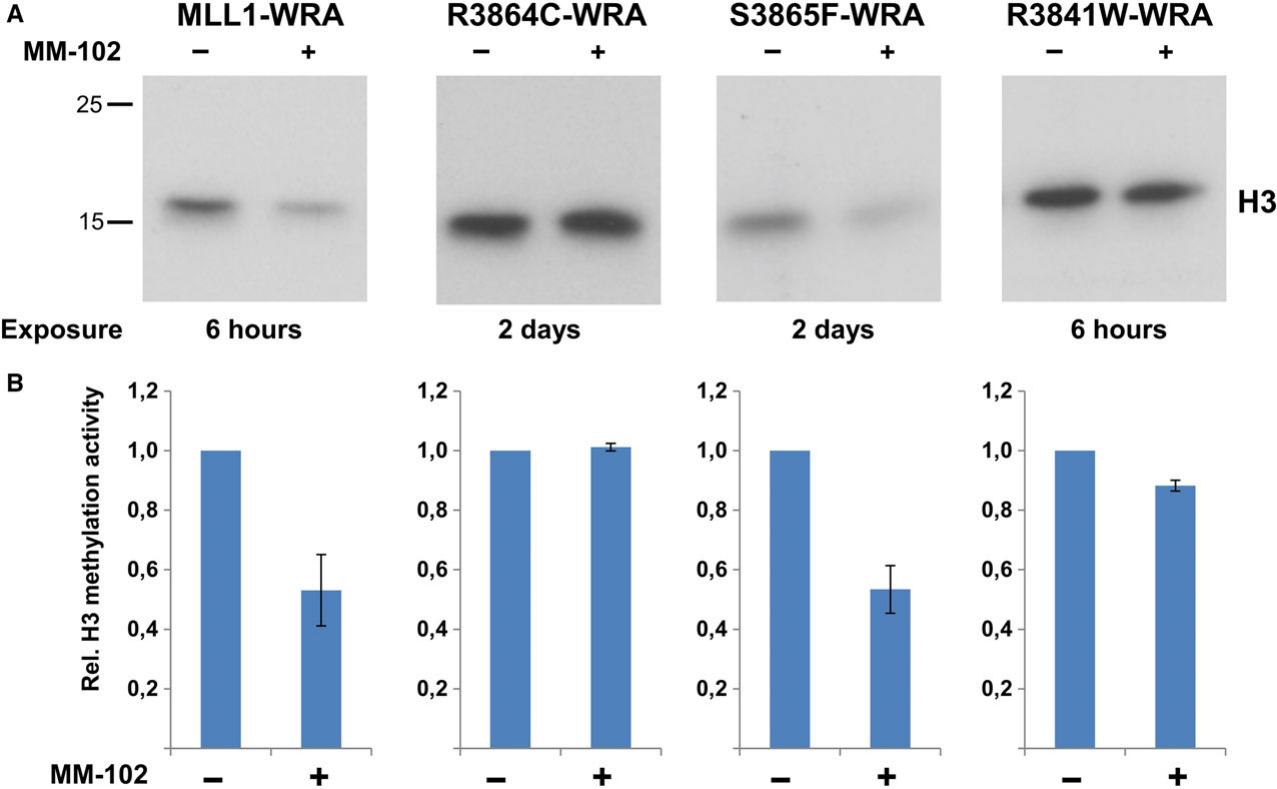
Inhibition of the MLL1 proteins by MM‐102. (A) Recombinant histone H3 was methylated by MLL1 cancer variants together with WRA complex in the presence and absence of inhibitor. Methylation signal of H3 and different exposure times are indicated. (B) Quantitative analysis of H3 methylation signals using duplicates of experiments. For better visualization of the inhibitory effect by MM‐102, the methylation activity of the different cancer variants was normalized to the corresponding sample without inhibitor treatment. The error bars indicate the standard error of the mean.

As MLL1 mutants exhibited differential methylation activities in the absence and presence of the WRA complex, we next tested the inhibition of the MLL1 mutant WRA complexes by the WDR5 binding inhibitor MM‐102. MLL1 wild‐type and the corresponding mutant proteins were incubated with the WRA complex proteins in the presence and absence of the inhibitor. The activity was tested as described above using histone H3 protein as methylation substrate and radioactively labeled AdoMet (Fig. 7). As reported (Karatas *et al*., 2013), MM‐102 efficiently inhibited the activity of the wild‐type MLL1. Inhibition was also observed for S3865F, which agrees with the finding that this mutant is dependent on the WRA complex to exhibit its full methyltransferase activity. In contrast to this and in agreement with the biochemical data, no inhibition was observed with the R3864C and R3841W variant proteins. This result was expected, because the inhibitor selectively disrupts the MLL1–WRA complex, but the activity of these two mutants was not stimulated by the WRA complex formation.

## 4 Discussion

Mixed lineage leukemia family PKMTs introduce H3K4 methylation and have important connections to cancer. They interact with the WDR5, RBBP5, and ASH2L (WRA) core complex partners together with additional associated subunits. Recently, Li *et al*. (2016) showed that MLL proteins primarily interact with the RBBP5/ASH2L heterodimer (RA heterodimer) and the interaction with WDR5 serves as a bridge to enhance RA binding (Li *et al*., 2016). Compared to other MLL family members, MLL1 is particularly dependent on the presence of WDR5 to exhibit full methyltransferase activity, because its binding to the RA heterodimer is weaker and WDR5 is needed as a bridge between MLL1 and the RA complex (Cao *et al*., 2014; Li *et al*., 2016).

Over the last years, it has been discovered that somatic missense mutations in several PKMTs such as EZH2, GLP, NSD2, MLL3, and MLL1 occur in cancer tissues and promote carcinogenesis by altering the catalytic activity or overall properties of the PKMT, including their activity, product pattern or substrate specificity (Kudithipudi and Jeltsch, 2014; Weirich *et al*., 2015; Yap *et al*., 2011). By Jan. 2017, the COSMIC database lists 23 MLL1 SET domain residues with somatic cancer mutations. At the time of the design of this study, we selected four somatic mutations in the SET domain of MLL1, which were observed in different cancers, and are positioned close to the active site or at the putative interfaces with WRA complex partners. Our data show that all of them influence the catalytic properties of MLL1 in a characteristic and distinct manner. Two somatic cancer mutants, R3864C and R3841W, exhibited differential catalytic properties and also displayed an altered response to the presence of the WRA complex partners. Both mutants were more active than wild‐type MLL1 in isolated form (R3841W approximately twofold and R3864C approximately 1.5‐fold), but their activity was not further stimulated in complex with the WRA proteins. This indicates a loss of the endogenous regulation of MLL1 activity in these mutants, because they are no longer controlled by the WRA complex. In contrast, two other mutants, S3865F and R3903H, showed a reduction (or complete loss) of activity. Hence our study in MLL1 provides examples of all classic mechanisms of oncogenic mutations in enzymes, loss of activity, hyperactivity and loss of regulation.

The molecular mechanism of the loss or reduction of activity of S3865F and R3903H can be deduced from the structural analysis of MLL1 (Li *et al*., 2016). Arginine 3903 is connecting the SET‐I and SET‐C domains suggesting that it participates in the pathway connecting conformational changes of SET‐I with catalytic activity. Our data indicate that the interactions of the R3903H mutant with the RA heterodimer and WDR5 are intact, but the exchange of arginine to histidine at the interface may alter the conformation of this critical region leading to the loss of activity. The critical role of this residue is supported by the finding that an R3903T mutant was also inactive (Shinsky *et al*., 2014) and the residue is fully conserved in all MLL enzymes.

S3865F is located in the loop contacting ASH2L. As in the case of R3903H, the interaction of S3865F with the WRA proteins is not disturbed, but catalytic activity is reduced, likely by an allosteric mechanism through which this loop affects the active site conformation. While Ser is only conserved at this site within the MLL1/2/TRX subfamily of MLL enzymes, all MLL enzymes contain a hydrophilic residue at this place (Ser, Thr or Gln). The reduction of activity of S3865F could therefore be related to the drastic change of a small hydrophilic residue (Ser) to a large aromatic one (Phe). The serine hydroxyl contributes to a stabilizing hydrogen bond network within the SET‐I subdomain and its replacement by a large hydrophobic residue that also faces the substrate binding pocket is bound to have an effect on activity. The interfaces with ASH2L and RBBP5 are not directly affected, which explains that the regulatory mechanisms via complex formation are not altered.

The changes induced by the R3864C and R3841W mutations can be interpreted in light of the specific effects of the WRA subcomplexes on the activity of the mutants and wild‐type MLL1 observed after screening of all possible combinations of complex partners. R3864 points toward ASH2L and RBBP5 in the MLL1 complex structure, where it is involved in an extensive electrostatic and hydrogen bonding network of interactions. The R3864C mutant reaches its maximal activity without complex partners, suggesting that the mutation induces a local conformational change of the SET‐I region which brings the active site into a closed conformation similar to other SET domain‐containing PKMTs. In this case, complex partners are not necessary to induce this conformational change and achieve full methyltransferase activity. The addition of RBBP5 or RA strongly inhibits the mutant, suggesting that the stimulatory effect is lost and an inactive conformation is adopted. The addition of WDR5, that is, complex formation with WRA, can partially compensate the loss of activity caused by RA, but even in the WRA complex R3864C is less active than without complex partners.

R3841 is located close to the active center forming a main‐chain H‐bond to AdoMet. Hence, it is located in the center of the region undergoing conformational changes in the MLL1 SET domain. Our data show that the R3841W is more active than wild‐type MLL1 without complex partners. Akin to R3864C, it is inhibited by the addition of RBBP5, but also by ASH2L in different combinations. WDR5 does not cause inhibition, and the activity of R3841W in the presence of WRA is also similar to the isolated enzyme. The resemblance of the profiles of R3864C and R3841W suggests that similar conformational changes are triggered by both mutations, one acting in the SET‐I and the other in the SET‐N part of the structure close to the active center. The results of our circular dichroism structure analyses support the notion that R3841W is folded but it shows a conformational difference to the wild‐type enzyme. While for detailed explanations of the structural rearrangements in the R3864C and R3841W mutants further experiment are necessary, our data clearly show that the activity of both MLL1 mutants is no longer regulated by the WRA complex.

Recently, the MM‐102 drug has been introduced as specific MLL1 inhibitor, which disrupts the interaction between MLL1 and WDR5 by mimicking the GSARAE residues of the Win motif in MLL1. It was shown to be an efficient inhibitor of MLL1 activity leading to a reduction of the expression of MLL1 target genes, such as HoxA9 and Meis1, in leukemia cell lines (Karatas *et al*., 2013). However, we show here that the activity of the R3864C and R3841W MLL1 mutants is not stimulated by complex formation with the WRA proteins. Consequently, MM‐102 does not have an inhibitory effect on the activity of these mutants, indicating that MM‐102 is a less promising therapeutic option in cancers bearing these MLL1 mutations. These data illustrate that the efficacy of inhibitors on mutant PKMTs must be experimentally confirmed before treatment is advisable. Conversely, mutant proteins may present novel targets allowing the development of specific drugs for cancer treatment.

## 5 Conclusions

Our data show that MLL1 mutations found in different tumors can stimulate or inhibit MLL1 activity indicating that MLL1 mutations act through cancer‐specific and variable molecular mechanisms. Moreover, two of the mutants have lost the natural control of MLL1 activity by the WRA complex. Hence, depending on the tumor type, inhibition of MLL1 or its hyperactivity and loss of regulation can promote tumor formation illustrating the complex and multifaceted role of MLL1 in cell fate determination and gene regulation. Our data exemplify that dedicated biochemical investigations are needed for each somatic tumor mutation of important proteins to decipher its pathological role. Furthermore, our data illustrate the relevance of the investigation of the effects of tumor mutations for cancer therapy. MM‐102 was shown to inhibit the interaction of MLL1 and WDR5 and act as an efficient and specific inhibitor of MLL1 activity. However, we show here that the activity of the R3864C and R3841W MLL1 mutants is not stimulated by complex formation with the WRA proteins. Consequently, MM‐102 does not have an inhibitory effect on these mutants, indicating that this inhibitor is a less powerful therapeutic option in cancers bearing these MLL1 mutations. These data illustrate that the efficacy of inhibitors of mutant PKMTs (or other mutant enzymes) must be experimentally validated before treatment.

## Availability of data and material

All data generated or analyzed during this study are included in this published article.

## Author contributions

AJ and SK devised the study. SW conducted the experiments with the help of SK. All authors were involved in data analysis and interpretation and preparation of the manuscript.

## Supporting information


**Table S1.** Compilation of *P*‐values for the data shown in this manuscript.
