# Supplementary material for: Somatic cancer mutations in the MLL1 histone methyltransferase modulate its enzymatic activity and dependence on the WDR5/RBBP5/ASH2L complex
Source: Mol Oncol. 2017 Mar 10;11(4):373–87. doi: 10.1002/1878-0261.12041 (PMC5527479; doi:10.1002/1878-0261.12041)
Supplement: Supplementary file 1 — Table S1. Compilation of P‐values for the data shown in this manuscript. [file MOL2-11-373-s001.pdf]

Somatic cancer mutations in MLL1 induce conformational changes and modulate the enzymatic activity

Sara Weirich, Srikanth Kudithipudi, & Albert Jeltsch*

Supplemental Table 1

Compilation of p-values for the data shown in this manuscript. For Figs. 2, 3, 5 and 6, p-values are based on average and standard deviation of normalized data (assuming a normal distribution). For Fig. 4, p-values were determined by a two sided Ttest assuming equal variance.

Data set	Comparison	p-value
Fig. 2C	wt / R3864C	<1e-10
	wt / S3865F	<1e-10
	wt / R3841W	<1e-10
	wt / R3903H	<1e-10
Fig. 2D	MLL1 / MLL1-WRA	<1e-10
Fig. 3B	MLL1 / MLL1-WRA	<1e-10
	wt / R3864C	<1e-10
	wt / R3864C-WRA	<1e-10
	wt / S3865F	<1e-10
	wt / S3865F-WRA	<1e-10
	wt / R3841W	<1e-10
	wt / R3841W-WRA	7.56E-05
	wt / R3903H	<1e-10
	wt / R3903H-WRA	<1e-10
Fig. 4B	wt-RA / R3864C-RA	7.93E-04
	wt-RA / S3865F-RA	3.60E-04
	wt-RA / R3841W-RA	2.18E-02
	wt-RA / GST control	5.42E-07
	wt-RA / only beads	6.31E-08
	wt-WRA / R3864C-WRA	1.55E-01
	wt-WRA / S3865F-WRA	2.19E-02
	wt-WRA / R3841W-WRA	1.04E-04
	wt-RA / wt-WRA	6.51E-07
	R3864C-RA / R3864C-WRA	5.69E-08
	S3865F-RA / S3865F-WRA	9.58E-06
	R3841W-RA / R3841W-WRA	3.20E-07

Data set	Comparison	p-value
Fig. 5C	wt / wt-A	0.376
	wt / wt-R	3.07E-03
	wt / wt-W	0.273
	wt / wt-AW	0.392
	wt / wt-RW	0.273
	wt / wt-AR	0.0111
	wt / wt-WRA	3.62E-07
Fig. 5C	R3864C / R3864C-A	0.193
	R3864C / R3864C-R	<1e-10
	R3864C / R3864C-W	0.0738
	R3864C / R3864C-AW	9.87E-05
	R3864C / R3864C-RW	<1e-10
	R3864C / R3864C-AR	3.62E-09
	R3864C / R3864C-WRA	1.46E-04
Fig. 5C	S3865F / S3865F-A	0.351
	S3865F / S3865F-R	2.95E-04
	S3865F / S3865F-W	0.334
	S3865F / S3865F-AW	7.09E-10
	S3865F / S3865F-RW	0.0757
	S3865F / S3865F-AR	0.364
	S3865F / S3865F-WRA	<1e-10
Fig. 5C	R3841W / R3841W-A	<1e-10
	R3841W / R3841W-R	<1e-10
	R3841W / R3841W-W	<1e-10
	R3841W / R3841W-AW	<1e-10
	R3841W / R3841W-RW	<1e-10
	R3841W / R3841W-AR	<1e-10
	R3841W / R3841W-WRA	<1e-10
Fig. 6B	wt-WRA +/- inhibitor	4.65E-05
	R3864C-WRA +/- inhibitor	0.188
	S3865F-WRA +/- inhibitor	2.86E-09
	R3841W-WRA +/- inhibitor	<1e-10
